# Not so Element-ary: A Copper Conundrum

**DOI:** 10.7759/cureus.9950

**Published:** 2020-08-23

**Authors:** Muhammad Asghar, Uzair Khan, Emily Horvath, Arsalan Khan

**Affiliations:** 1 Internal Medicine, University of Illinois College of Medicine at Peoria, Peoria, USA; 2 Clinical Nutrition, OSF Healthcare, Peoria, USA

**Keywords:** wilson disease, copper, duodenal switch

## Abstract

Copper is an essential micronutrient required for a number of enzymatic activities. Deficiency is relatively rare as only trace amounts are needed to maintain homeostasis. Deficiencies however do occur and are most commonly seen in malabsorptive states such as after bariatric surgeries. Herein, we present an interesting case of copper deficiency diagnosed in a 70-year-old male patient post duodenal switch procedure which persisted despite aggressive oral and intravenous copper supplementation. This lack of response to supplementation prompted further evaluation, leading to a diagnosis of underlying heterozygous Wilson's disease.

## Introduction

Copper is an essential element ubiquitous in nature required only in trace amounts. The daily copper requirement is 0.9 mg/dl usually met with the dietary sources [1]. It is vital for the functioning of cuproenzymes, which use it as a cofactor in respiration, neurotransmitter synthesis, and connective tissue formation. Copper deficiency though rare has been described in the setting of malabsorptive states such as bariatric surgery or short bowel syndrome. Copper deficiency has a large variation in clinical presentation from hematologic to neurologic, while copper excess leads to liver cirrhosis [2]. We describe a case of incidental low serum copper concentration in a male with a history of duodenal switch, a form of gastric bypass surgery. We also briefly review the diagnosis of copper deficiency with emphasis on heterozygous Wilson’s as a differential for low serum copper concentration.

## Case presentation

Our patient was a 70-year-old male with a history of refractory bile acid reflux for which he underwent a duodenal switch in 2012. He presented initially to his primary care physician for workup due to gum hypertrophy. He has had no changes in weight and no reported vitamin or mineral deficiencies since his surgery. His medical history is significant for gastroesophageal reflux, benign prostatic hyperplasia, atrial fibrillation, asthma, hypertension, and anxiety. His past surgical history is significant for only the aforementioned duodenal switch procedure. His weight was 74 kg and height 1.80 meters with no significant changes in weight recently. On history, he was noted to eat three meals daily of ostensibly normal portions and was not following any restrictive diets. His medication list was as follows: zolpidem 10 mg, atorvastatin 20 mg, lorazepam 0.5 mg, omeprazole 20 mg, flecainide 50 mg, carvedilol 25 mg, losartan 50 mg, budesonide-formoterol inhaler, gabapentin 300 mg twice daily, and apixaban 5 mg tab. His serum copper concentration at initial presentation were 0.7 mcg/ml (0.7 - 1.45). Complete blood count (CBC), comprehensive metabolic panel (CMP) zinc, vitamin A, D, and B12 concentrations were normal (Table 1). Since the patient was asymptomatic, he was recommended dietary modifications with periodic monitoring of copper concentration.

**Table 1:** Serum copper concentration at initial visit and trends over the course of treatment; normal values of zinc, thiamine, folate, vitamin D-OH, iron, and ferritin can also be seen

Table 1	Normal Range	9/13/17	12/15/17	8/21/18	9/29/18	11/6/18	12/16/18	1/1/19
Serum Copper	0.75-1.50 mcg/mL	0.60	0.70	0.69	0.56	0.57	0.39	0.29
Urinary copper	<=60 mcg/24 hours	-	-	-	-	-	39	28
Serum Ceruloplasmin	20-60 mg/dL	n/a	-	-	-	-	10	-
Serum Zinc	0.66-1.10 mcg/mL	n/a	0.87	0.85		1.11	0.76	0.92
Thiamine	70-180 nmol/L	168	-	-	-	-	-	-
Folate	7.0-31.4 ng/mL	>40	-	-	-	-	-	-
Vitamin D-OH	20-40 ng/mL	40	-	-	-	-	-	-
Iron	31-144 mcg/dL	n/a	-	-	-	-	-	-
Total iron binding capacity (TIBC)	261-462 mcg/dL	n/a	-	-	-	-	-	-
Iron % saturation	15-62%	42%	-	-	-	-	-	-
Ferritin	22-274 ng/mL	128	-	-	-	-	-	-

Repeat laboratory work done after six months showed copper concentration to be 0.69 mcg/ml with serum ceruloplasmin of 16 (20-60 mg/dL). Copper replacement regimen was started with oral copper supplementation of 4 mg/day.

Upon three months follow up after copper supplementation, his serum copper concentration had further dropped to 0.5 mcg/ml and ceruloplasmin to 14. Other minerals and vitamin levels were tested again. Zinc, manganese, iron, molybdenum, selenium, chromium and Vitamin A, D, E, K all were normal. Oral copper supplements were increased to 8 mg/day and a repeat copper concentration was performed at one month which showed a further drop in serum copper to 0.42 mcg/ml with ceruloplasmin of 10 mg/dl.

At this point, the patient was admitted for further workup and was started on Intravenous copper infusion at a rate of 2.4 mg CuCl/day. Serum copper continued to drop down to 0.30 mcg/ml. Ceruloplasmin was 11 mg/dl. Despite increasing the infusion rate to 4 mg over eight hours at night, serum copper and ceruloplasmin concentrations remained low at 0.29 mcg/ml and 10 mg/dl respectively.

**Figure 1:**
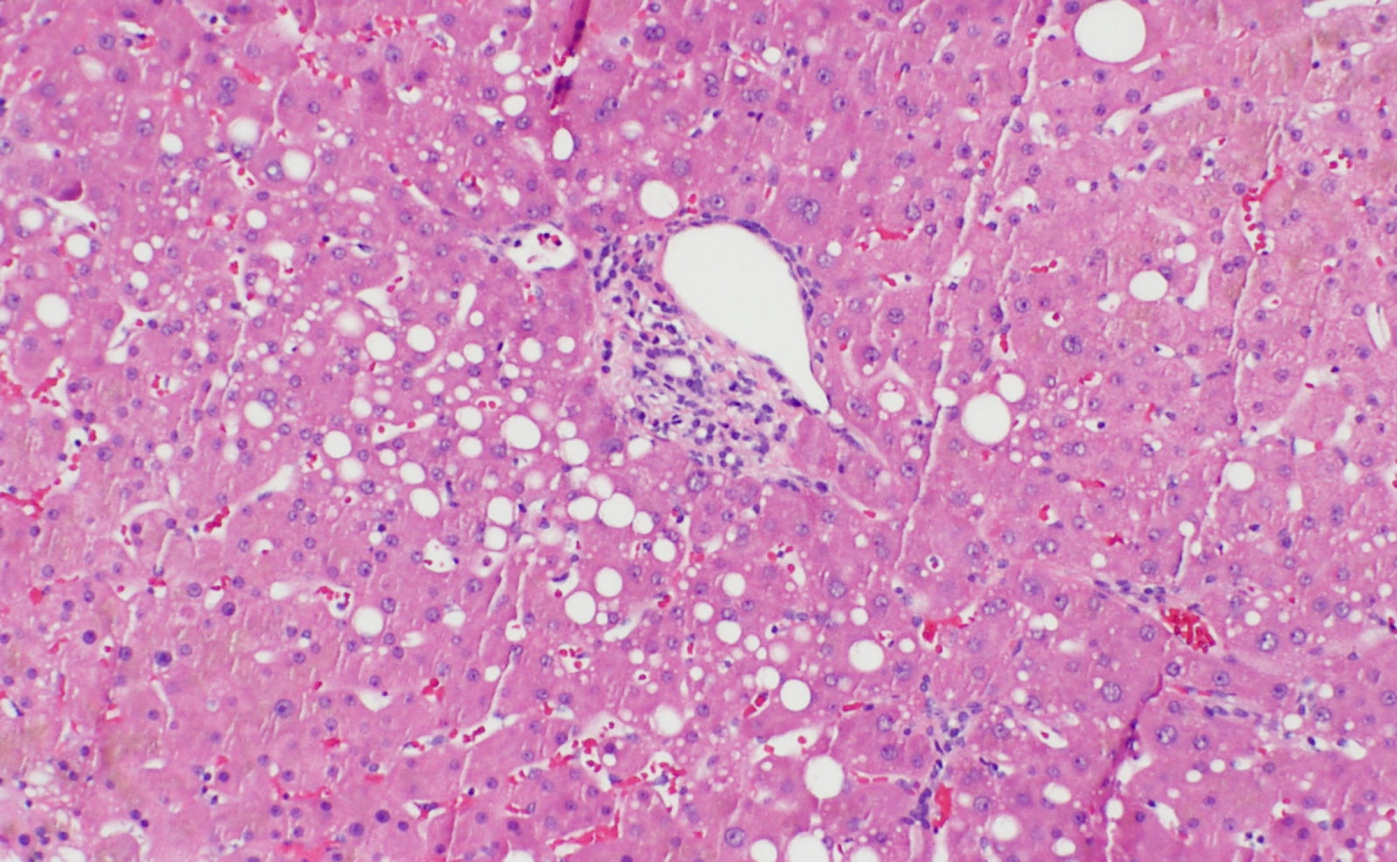
Liver biopsy showing mild macrovesicular steatosis and cholestasis

The rest of the patient's workup including CBC, CMP, and CT scan were unremarkable. At this point, a liver biopsy was performed which showed liver with mild macrovesicular steatosis and cholestasis (Figure 1).

Liver copper concentration was 187 mcg/g, which although above normal, was less than 225 mcg/g cutoff normally seen in Wilson's disease. This above-normal concentration prompted further testing for Wilson's disease which ultimately showed the patient to be heterozygous. Gene testing came back positive for DNA mutation 3402de1C and amino acid change A113SQfs*13. This alteration is a pathogenic mutation for heterozygous Wilson's disease. IV copper infusion was stopped after the diagnosis and labs stabilized over the following 2-3 weeks. Since then our patient has been without copper supplementation and has no reported physical or biochemical symptoms of copper deficiency. In the setting of being heterozygous for Wilson’s disease, no further serum copper testing has been drawn.

## Discussion

The most widely used biomarker for copper status is serum copper. However, care should be taken with its interpretation since it does not always reliably reflect body stores. A systematic review of copper biomarkers found that low serum copper concentration may not respond significantly to supplementation [3]. This lack of response to supplementation is due to the fact that serum copper is bound to ceruloplasmin, which is itself an unreliable marker in inflammatory/stressed states [3,4]. Alternate biomarkers of copper, such as platelet copper, erythrocyte copper, and erythrocyte superoxide dismutase, have all been shown to be as unreliable as serum copper and are not routinely used in assessing copper deficiency [3,4]. The gold standard for the assessment of total body copper is liver biopsy with staining but its invasiveness limits its routine use. Our subject’s paradoxical reaction to copper supplementation perfectly highlights the dangers of ‘chasing numbers’ and shows the complexity in the interpretation of serum copper concentrations especially in the setting of disturbed copper homeostasis such as Wilson’s disease.

Wilson’s disease is an autosomal recessive disease with a prevalence of 30 per million of the population worldwide. The genetic defect is localized to chromosome 13 (ATP7B), the gene responsible for copper transport is adenosine triphosphatase (ATPase) [1,5]. Heterozygous Wilson's disease involves two different mutations and has a prevalence of 1:90. Usually, heterozygous Wilson's disease patients have normal serum copper and ceruloplasmin levels and rarely have clinical symptoms due to incomplete penetrance; only 20% have significantly lower levels than normal individuals. 

Wilson’s disease carriers have defective ATPase activity and are postulated to have half the number of ATP7B transporters resulting in decreased copper transport to ceruloplasmin [6,7]. Ceruloplasmin lacking copper (apoceruloplasmin) is quickly degraded in the bloodstream leading to low serum ceruloplasmin. Supplementation with oral and intravenous copper in heterozygous Wilson's disease patients to replenish low serum copper concentration in the setting of duodenal switch procedure or any other malabsorptive state can result in further down-regulation of ATP7B activity. Decreased ATP 7B activity with supplementation impairs excretion via bile resulting in increased total body copper retention and deposition in different organs giving characteristic findings [7]. 

Copper deficiency in our patient was also initially thought to be due to duodenal switch [8,9]; however, it was further complicated by his heterozygote Wilson’s status impairing transport of copper. This was reflected by continued low serum copper concentration despite supplementation. This prompted further workup leading us to the diagnosis of Wilson's disease. Had we missed the diagnosis and continued supplementation, it could have led to irreversible neurologic defects. 

## Conclusions

Our case is unique as low copper concentration in our patient was due to Wilson's disease rather than his malabsorptive state from his duodenal switch. Hence, care should be taken in ‘treating numbers’; if serum copper and ceruloplasmin concentrations do not respond to adequate supplementation, alternate diagnoses such as Wilson's disease should be on the differential.
